# Prognostic role of fibrinogen-to-albumin ratio in patients with gynecological cancers: a meta-analysis

**DOI:** 10.3389/fonc.2025.1580940

**Published:** 2025-07-03

**Authors:** Yaping Chen, Jiliang Zhang

**Affiliations:** ^1^ Clinical Laboratory, Maternal and Child Health Hospital of Changxing County, Huzhou, Zhejiang, China; ^2^ Clinical Laboratory, Lishui People’s Hospital, The Sixth Affiliated Hospital of Wenzhou Medical University, Lishui, Zhejiang, China

**Keywords:** fibrinogen-to-albumin ratio, meta-analysis, gynecological cancers, prognosis, evidence-based medicine

## Abstract

**Background:**

Fibrinogen-to-albumin ratio (FAR) has been widely studied for its prognostic value in gynecological cancers, but the results remain inconsistent. Therefore, this study aimed to evaluate the precise prognostic significance of FAR in gynecological cancers.

**Methods:**

A comprehensive literature search was conducted in PubMed, Web of Science, Embase, the Cochrane Library, and China National Knowledge Infrastructure (CNKI) databases up to 12 May 2025. The prognostic value of FAR for overall survival (OS) and progression-free survival (PFS) in gynecological cancers was examined using pooled hazard ratios (HRs) and corresponding 95% confidence intervals (CIs).

**Results:**

A total of 10 articles comprising 1,902 patients were included in this meta-analysis. Pooled results indicated that elevated FAR was significantly associated with poor OS (HR = 2.75, 95% CI: 2.26–3.36, *p* < 0.001) and shorter PFS (HR = 1.60, 95% CI: 1.20–2.12, *p* = 0.001) in patients with gynecological cancers. Subgroup analyses confirmed that FAR predicted OS regardless of sample size, cancer type, FIGO stage, treatment modality, FAR threshold, threshold determination method, or type of survival analysis (*p* < 0.05). Additionally, FAR remained a significant predictor of poor PFS across different cancer types.

**Conclusion:**

This meta-analysis showed that a high FAR is significantly associated with worse OS and PFS in patients with gynecological cancers. FAR may serve as a promising prognostic biomarker in clinical practice.

**Systematic review registration:**

https://inplasy.com/inplasy-2025-5-0036/, identifier INPLASY202550036.

## Introduction

Gynecological cancers, the most prevalent female malignancies globally, affect the reproductive system and significantly impact the quality of life (1). These cancers typically include endometrial, cervical, vulvar, ovarian, and vaginal cancers (2). They can impair reproductive organ function, leading to negative effects on sexual health, self-concept, self-esteem, and physical fitness (3). According to GLOBOCAN, 1,471,803 new cases of gynecological cancers and 679,549 cancer-related deaths were recorded in 2022 globally (2). Despite major advancements in surgical, radiation, and chemotherapy treatments, the prognosis for advanced-stage gynecological cancers remains poor due to limited treatment options. The 5-year survival rates for cervical, endometrial, and ovarian cancers are only 17.2%, 16.3%, and 29.2%, respectively (4, 5). Prognostic markers play a crucial role in improving survival outcomes in patients with gynecological cancers (6). Therefore, identifying reliable and effective prognostic markers is essential.

Increasing data show that deficiencies in nutrition, hemostatic elements, and inflammation are key contributors to the development of human cancers (7). Previous studies have identified several hematological parameters that are crucial biomarkers for cancer prognosis, including platelet-to-lymphocyte ratio (8), prognostic nutritional index (9), systemic immune inflammation index (10), and albumin-to-globulin ratio (11). The fibrinogen-to-albumin ratio (FAR), obtained based on routine blood test results as FAR = fibrinogen/albumin, has also demonstrated significant potential in predicting cancer prognosis. FAR has been linked to outcomes in various cancers, including laryngeal cancer (12), non-small-cell lung cancer (13), diffuse large B-cell lymphoma (14), pancreatic cancer (15), and hepatocellular carcinoma (16). Although many studies have explored the prognostic value of FAR in patients with gynecological cancers, the findings remain inconsistent (17–26). Some studies reported that elevated FAR is markedly associated with poor prognosis in gynecological cancers (17, 19, 22, 23), while others suggest that a higher FAR is linked to improved survival outcomes (24). Therefore, we conducted this meta-analysis to identify the accurate prognostic value of FAR in gynecological cancers.

## Materials and methods

### Study guideline

The study was conducted in strict accordance with the Preferred Reporting Items for Systematic Reviews and Meta-Analyses (PRISMA) guidelines (27). The research protocol was registered in INPLASY (registration number: INPLASY202550036), and the protocol is accessible at https://inplasy.com/inplasy-2025-5-0036/.

### Search strategy

We systematically searched PubMed, Web of Science, Embase, the Cochrane Library, and China National Knowledge Infrastructure (CNKI) up to 12 May 2025. The search strategy included the following terms: (albumin-to-fibrinogen OR albumin/fibrinogen OR fibrinogen-to-albumin OR fibrinogen/albumin) AND (cervical cancer OR gynecological cancer OR ovarian cancer OR endometrial cancer OR gynecological carcinoma OR cervical carcinoma OR ovarian carcinoma OR endometrial carcinoma OR gynecological neoplasm OR vulvar cancer OR vaginal cancer). No publication language restriction was applied. Detailed search strategies for each database are provided in **Supplementary File 1**. Moreover, reference lists of relevant studies were manually screened to identify any further eligible articles.

### Inclusion and exclusion criteria

Studies were included based on the following criteria (1): pathological diagnosis of gynecological cancers, including cervical, endometrial, ovarian, vulvar, and vaginal cancers (2); investigation of the association between FAR and survival outcomes in gynecological cancers (3); reporting of hazard ratios (HRs) with 95% confidence intervals (CIs) (4); identification of a FAR threshold; and (5) publication in any language. The exclusion criteria were (1): meeting abstracts, reviews, comments, letters, or case reports (2); studies with duplicate data; and (3) animal studies.

### Data extraction and quality assessment

Two investigators (Y.C. and J.Z.) independently extracted data from eligible articles, and any disagreements were settled through discussion. The following information was collected: author, year, country, sample size, age, study period, study design, study center, cancer type, International Federation of Gynecology and Obstetrics (FIGO) stage, treatment, FAR threshold, threshold determination method, follow-up duration, survival outcomes, survival analysis, HRs, and 95% CIs. Overall survival (OS) and progression-free survival (PFS) were considered the primary and secondary outcomes, respectively. Moreover, the quality of the included studies was evaluated using the Newcastle–Ottawa scale (NOS; range: 0–9 points) (28), with a score of ≥ 6 indicating high quality.

### Statistical analysis

We evaluated the prognostic value of FAR for predicting OS and PFS in gynecological cancers by computing pooled HRs and 95% CIs. Between-study heterogeneity was assessed using Cochran’s Q test and *I*-squared (*I* ^2^) statistic. A random-effects model was employed when significant heterogeneity was present (*I* ^2^ > 50%); otherwise, a fixed-effects model was used. Subgroup analyses were conducted to explore potential sources of heterogeneity. Sensitivity analysis was performed by sequentially excluding individual studies to evaluate the robustness of the pooled results. Publication bias was assessed using Begg’s and Egger’s tests. All statistical analyses were conducted using Stata software version 12.0 (Stata Corp, College Station, TX, USA). A *p* < 0.05 was considered statistically significant.

## Results

### Process of literature search

**Figure 1:**
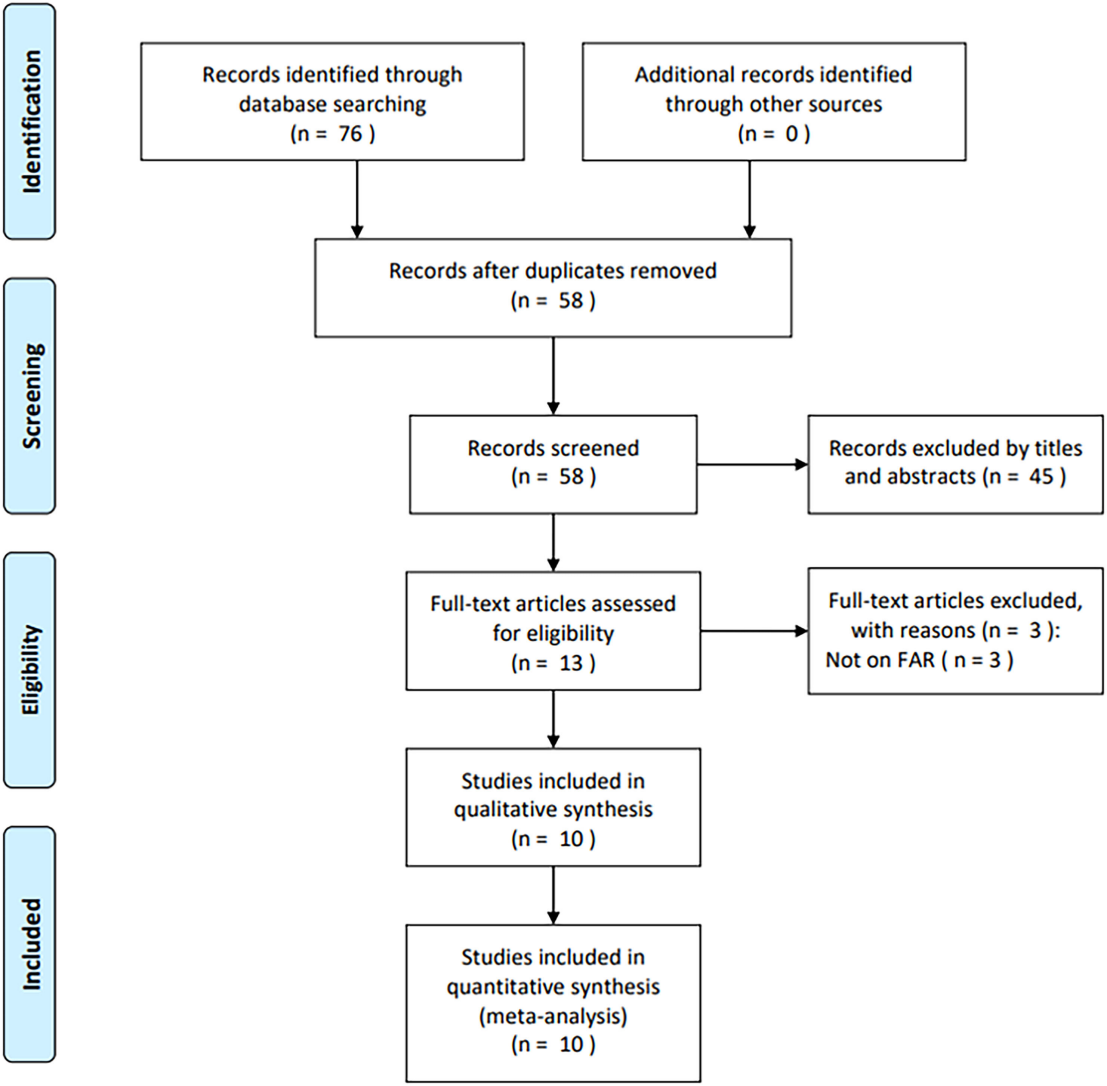
PRISMA flow chart illustrating the process of study screening.

A total of 76 studies were initially identified through primary database searches (PubMed, *n* = 18; Web of Science, *n* = 15; Embase, *n* = 29; Cochrane Library, *n* = 1; and CNKI, *n* = 13). After removing duplicates, 58 studies remained (**Figure 1**). Following a review of titles and abstracts, 45 records were excluded due to irrelevance. The full texts of the remaining 13 articles were then assessed, with three excluded for not reporting FAR (*n* = 3). Eventually, 10 articles comprising 1,902 cases (17–26) were included in the final analysis (**Figure 1**).

### Characteristics of included studies

The included articles were published between 2019 and 2024 (**Table 1**) and were retrospective in design (17–26). Eight studies were performed in China (17–23, 26), while two were carried out in Austria (24, 25). Sample sizes ranged from 59 to 342, with a median of 182.5. Five articles were published in English (17, 19, 23–25) and five in Chinese (18, 20–22, 26). Six studies involved patients with ovarian cancer (17, 18, 20, 23, 25, 26), three included patients with cervical cancer (19, 21, 22), and one focused on vulvar cancer (24). Regarding treatment modalities, six studies evaluated surgical cases (20–24, 26), three involved surgery followed by chemotherapy (18, 19, 25), and one study assessed neoadjuvant chemotherapy followed by surgery (17). The FAR thresholds ranged from 0.097 to 0.13, with a median of 0.11. Seven studies examined the prognostic value of FAR for OS (17–19, 21–23, 26), while another seven reported the association between FAR and PFS (17–20, 23–25) in gynecological cancers. Six articles reported HRs with 95% CIs from multivariate analyses (17–19, 21, 23, 26), while four employed univariate regression (20, 22, 24, 25). The NOS scores ranged from 7 to 9, indicating high study quality (17–26) (**Table 1**).

**Table 1:** Basic characteristics of the studies included in this meta-analysis.

Study	Year	Country	Sample size	Age (year) Median (range)	Study period	Tumor type	FIGO stage	Treatment	Cutoff value	Cutoff determination	Follow-up (month) Median (range)	Survival outcomes	Survival analysis	NOS score
Yu, W (17).	2019	China	313	65	2010–2017	OC	III–IV	NAC + surgery	0.129	ROC curve	1–80	OS, PFS	Multivariate	8
Zhang, W (18).	2019	China	342	50 (24–76)	2010–2013	OC	I–IV	Surgery + chemotherapy	0.11	Median value	37.5 (2–104)	OS, PFS	Multivariate	9
An, Q (19).	2020	China	278	45.5	2010–2017	CC	I–II	Surgery + chemotherapy	0.13	ROC curve	1–60	OS, PFS	Multivariate	8
He, Y (20).	2021	China	59	57 (27–83)	2013–2019	OC	I–IV	Surgery	0.101	ROC curve	56 (9–90)	PFS	Univariate	7
Liu, X (21).	2021	China	80	52 (28–76)	2017–2019	CC	I–II	Surgery	0.103	ROC curve	1–60	OS	Multivariate	8
Zhao, X (22).	2021	China	124	48 (30–70)	2014–2017	CC	I–IV	Surgery	0.104	ROC curve	1–36	OS	Univariate	7
Chen, W (23).	2022	China	114	54	2007–2018	OC	I–IV	Surgery	0.12	ROC curve	52 (1–170)	OS, PFS	Multivariate	8
Onoprienko, A (24).	2022	Austria	204	69 (58–79)	2000–2020	VC	I–IV	Surgery	0.097	Median value	1–60	PFS	Univariate	9
Postl, M (25).	2024	Austria	161	58 (50–67)	2006–2020	OC	II–IV	Surgery + chemotherapy	0.11	Median value	1–60	PFS	Univariate	8
Yang, X (26).	2024	China	227	49	2015–2021	OC	I–IV	Surgery	0.13	ROC curve	1–36	OS	Multivariate	8

*NAC*, neoadjuvant chemotherapy; *OC*, ovarian cancer; *CC*, cervical cancer; *VC*, vulvar cancer; *ROC*, receiver operating characteristic; *OS*, overall survival.

*PFS*, progression-free survival; *FIGO*, International Federation of Gynecology and Obstetrics; *NOS*, Newcastle-Ottawa Scale.

### FAR and OS

**Figure 2:**
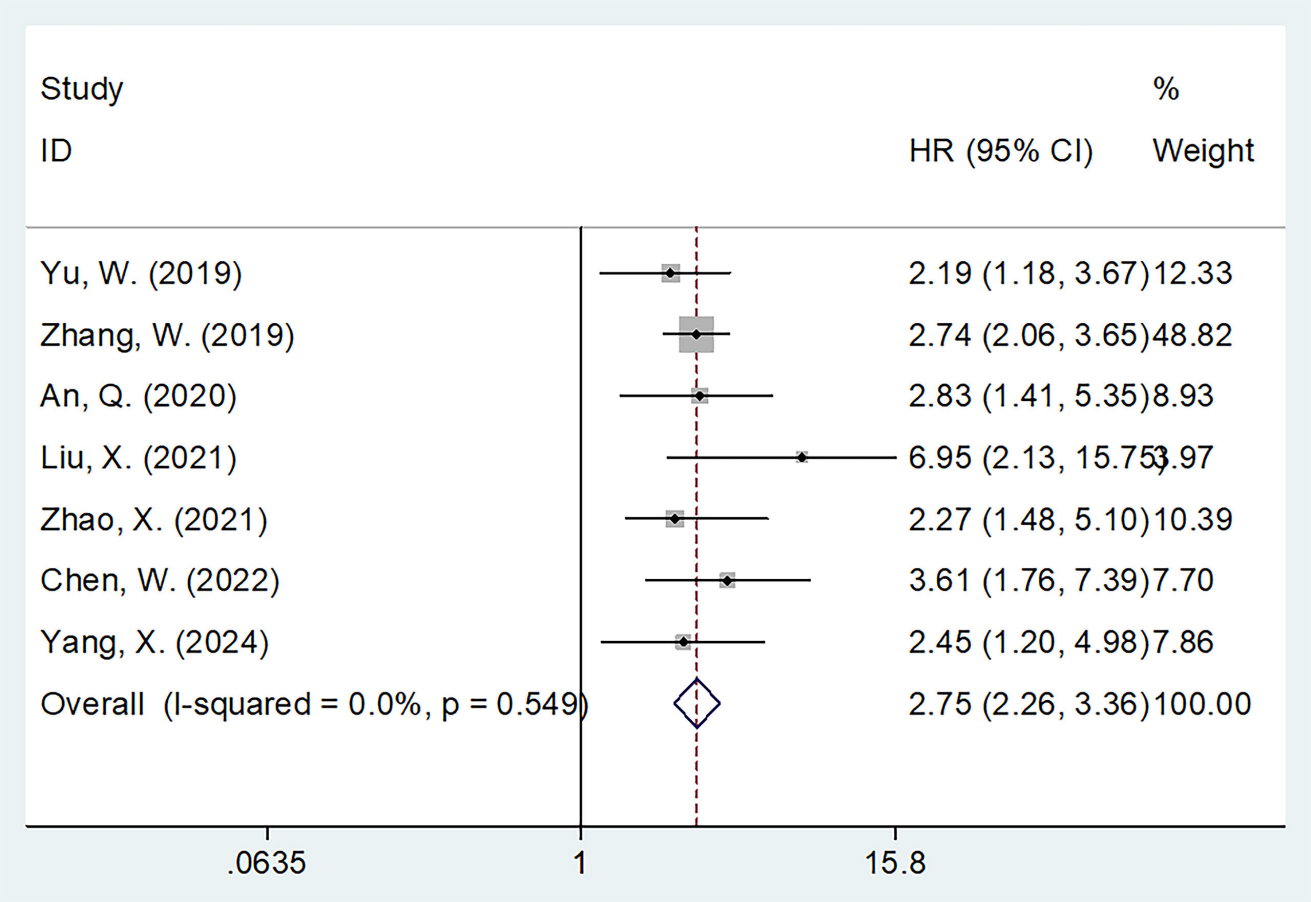
Forest plots showing the prognostic value of FAR for OS in patients with gynecological cancers.

Seven studies involving 1,478 patients (17–19, 21–23, 26) reported the association between FAR and OS in gynecological cancers. Heterogeneity among the studies was not significant (*I* ^2^ = 0, *p* = 0.549); therefore, a fixed-effects model was applied (**Table 2**). The pooled analysis revealed that a high FAR was a significant prognostic biomarker for poor OS in gynecological cancers (HR = 2.75, 95% CI: 2.26–3.36, *p* < 0.001; **Table 2**; **Figure 2**). Subgroup analyses further demonstrated that the prognostic value of FAR for OS remained consistent regardless of sample size, cancer type, FIGO stage, treatment modality, threshold, threshold determination method, or type of survival analysis (*p* < 0.05; **Table 2**).

**Table 2:** Subgroup analysis of the prognostic value of FAR for OS in patients with gynecological cancers.

Subgroups	No. of studies	No. of patients	Effects model	HR (95% CI)	*p*-value	Heterogeneity	
						*I* ^2^ (%)	pH
Total	7	1478	Fixed	2.75 (2.26–3.36)	< 0.001	0	0.549
Sample size							
< 200	3	318	Fixed	3.26 (2.13–4.98)	< 0.001	44.6	0.165
≥ 200	4	1,160	Fixed	2.63 (2.10–3.29)	< 0.001	0	0.903
Cancer type							
OC	4	996	Fixed	2.69 (2.14–3.37)	< 0.001	0	0.746
CC	3	482	Fixed	2.99 (1.98–4.52)	< 0.001	43.4	0.171
FIGO stage							
I–II	2	358	Random	4.09 (1.72–9.74)	0.001	53.5	0.143
I–IV	4	807	Fixed	2.71 (2.16–3.42)	< 0.001	0	0.798
II–IV/III–IV	1	313	–	2.19 (1.24–3.86)	0.001	–	–
Treatment							
Surgery	4	545	Fixed	3.02 (2.10–4.35)	< 0.001	26.3	0.254
NAC + surgery/surgery + chemotherapy	3	933	Fixed	2.65 (2.09–3.36)	< 0.001	0	0.768
Cutoff value							
< 0.11	2	204	Random	3.70 (1.24–10.98)	0.019	71.4	0.062
≥ 0.11	5	1,274	Fixed	2.70 (2.18–3.35)	< 0.001	0	0.870
Cutoff determination							
ROC curve	6	1,136	Fixed	2.77 (2.09–3.65)	< 0.001	0	0.421
Median value	1	342	–	2.74 (2.06–3.65)	< 0.001	–	–
Survival analysis							
Univariate	1	124	–	2.27 (1.22–4.21)	0.009	–	–
Multivariate	6	1,354	Fixed	2.82 (2.28–3.48)	< 0.001	0	0.476

*FAR*, fibrinogen-to-albumin ratio; *OS*, overall survival; *NAC*, neoadjuvant chemotherapy; *OC*, ovarian cancer; *CC*, cervical cancer; *ROC*, receiver operating characteristic; *FIGO*, International Federation of Gynecology and Obstetrics.

### FAR and PFS

**Figure 3:**
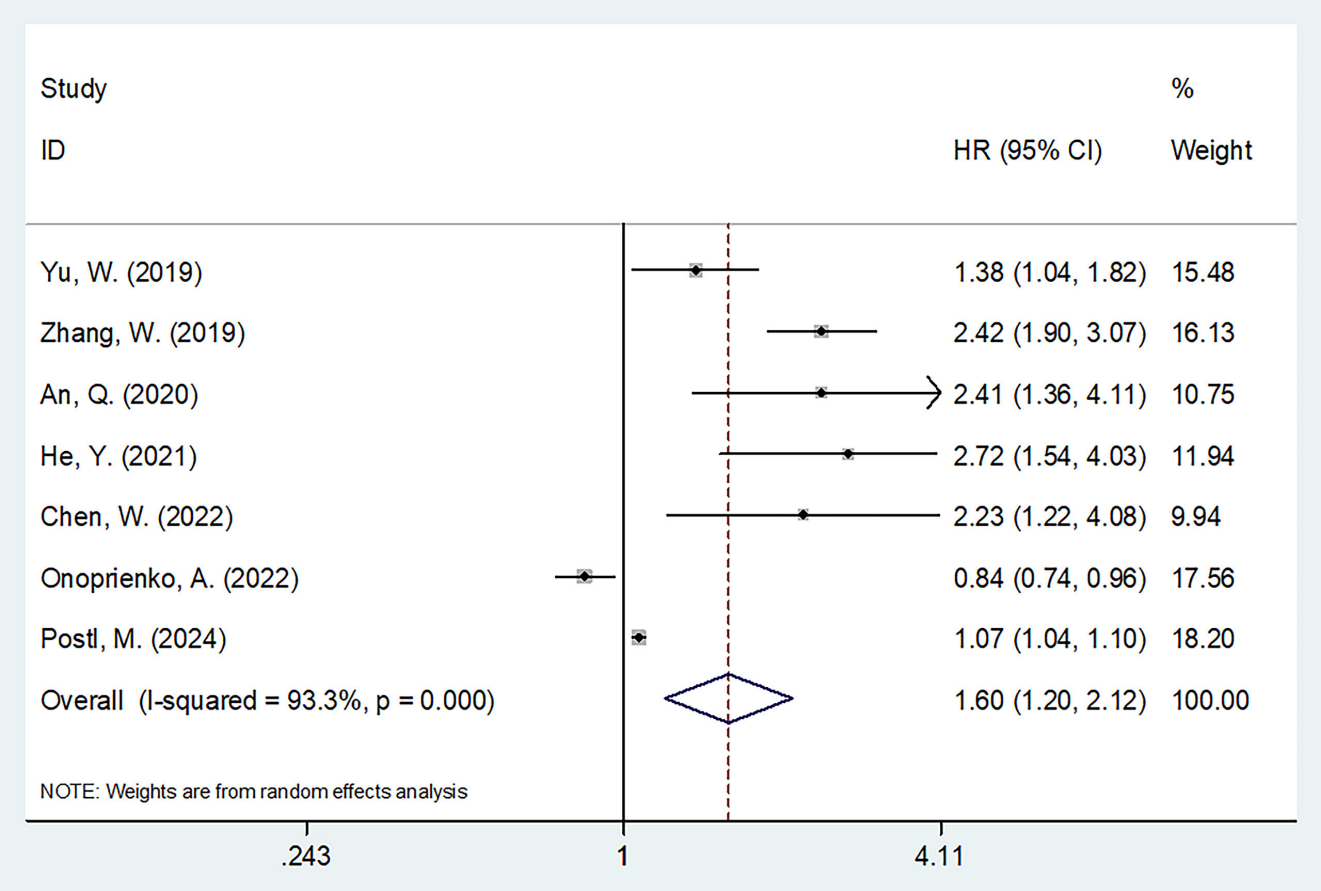
Forest plots showing the prognostic value of FAR for PFS in patients with gynecologic cancers.

Seven studies comprising 1,471 patients (17–20, 23–25) evaluated the correction between FAR and PFS. Due to significant heterogeneity (*I* ^2^ = 93.3%, *p* < 0.001), a random-effects model was applied. The pooled results showed that a higher FAR was significantly associated with poorer PFS in gynecological cancers (HR = 1.60, 95% CI: 1.20–2.12, *p* = 0.001; **Table 3**; **Figure 3**). Subgroup analyses confirmed that FAR consistently predicted worse PFS across different cancer types (**Table 3**).

**Table 3:** Subgroup analysis showing the prognostic value of FAR for PFS in patients with gynecological cancers.

Subgroups	No. of studies	No. of patients	Effects model	HR (95%CI)	*p*-value	Heterogeneity	
						*I* ^2^ (%)	pH
Total	7	1 471	Random	1.60 (1–20-2.12)	0.001	93.3	< 0.001
Country							
China	5	1,106	Random	2.11 (1.57–2.83)	< 0.001	64.4	0.024
Austria	2	365	Random	0.96 (0.75–1.21)	0.711	92.1	< 0.001
Sample size							
< 200	3	334	Random	1.80 (0.89–3.61)	0.100	90.0	< 0.001
≥ 200	4	1,137	Random	1.58 (0.87–2.86)	0.132	95.6	< 0.001
Cancer type							
OC	5	989	Random	1.79 (1.15–2.80)	0.010	93.9	< 0.001
CC	1	278	–	2.41 (1.39–4.19)	0.002	–	–
VC	1	204	–	0.84 (0.74-0.96)	0.009	–	–
FIGO stage							
I–II	1	278	–	2.41 (1.39–4.19)	0.002	–	–
I–IV	4	719	Random	1.84 (0.89–3.79)	0.099	96.1	< 0.001
II–IV/III–IV	2	474	Random	1.17 (0.92–1.48)	0.199	68.2	0.076
Treatment							
Surgery	3	933	Random	1.67 (0.70–4.02)	0.251	93.2	< 0.001
NAC + surgery/surgery + chemotherapy	4	538	Random	1.67 (1.04–2.67)	0.033	94.5	< 0.001
Cutoff value							
< 0.11	2	263	Random	1.48 (0.47–4.66)	0.507	95.3	< 0.001
≥ 0.11	5	1,208	Random	1.75 (1.13–2.69)	0.011	93.3	< 0.001
Cutoff determination							
ROC curve	4	764	Random	2.03 (1.39–2.94)	< 0.001	61.7	0.049
Median value	3	707	Random	1.27 (0.88–1.83)	0.208	96.5	< 0.001
Survival analysis							
Univariate	3	424	Random	1.17 (0.87–1.58)	0.291	92.7	< 0.001
Multivariate	4	1,047	Random	2.00 (1.43–2.81)	< 0.001	69.1	0.021

*FAR*, fibrinogen-to-albumin ratio; *PFS*, progression-free survival; *NAC*, neoadjuvant chemotherapy; *OC*, ovarian cancer; *CC*, cervical cancer; *VC*, vulvar cancer; *ROC*, receiver operating characteristic; *FIGO*, International Federation of Gynecology and Obstetrics.

### Sensitivity analysis

**Figure 4:**
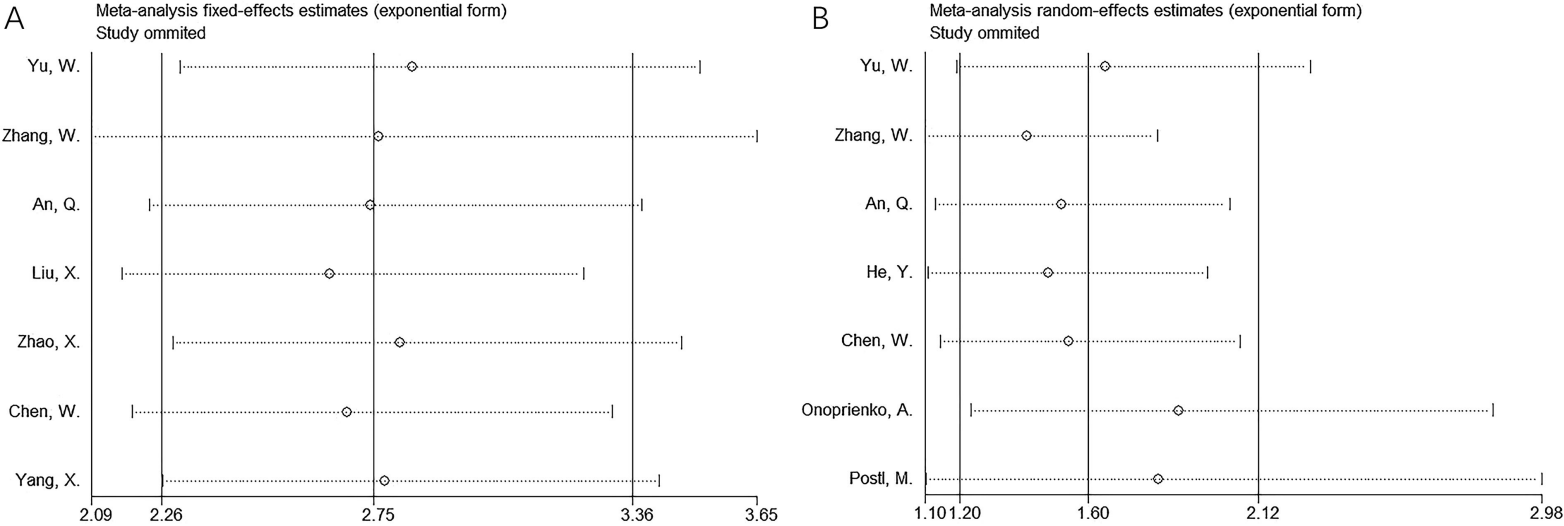
Sensitivity analysis for **(A)** OS and **(B)** PFS.

Sensitivity analysis was performed by sequentially removing each study, and no significant changes were observed in the pooled results for OS and PFS (**Figure 4**).

### Publication bias

**Figure 5:**
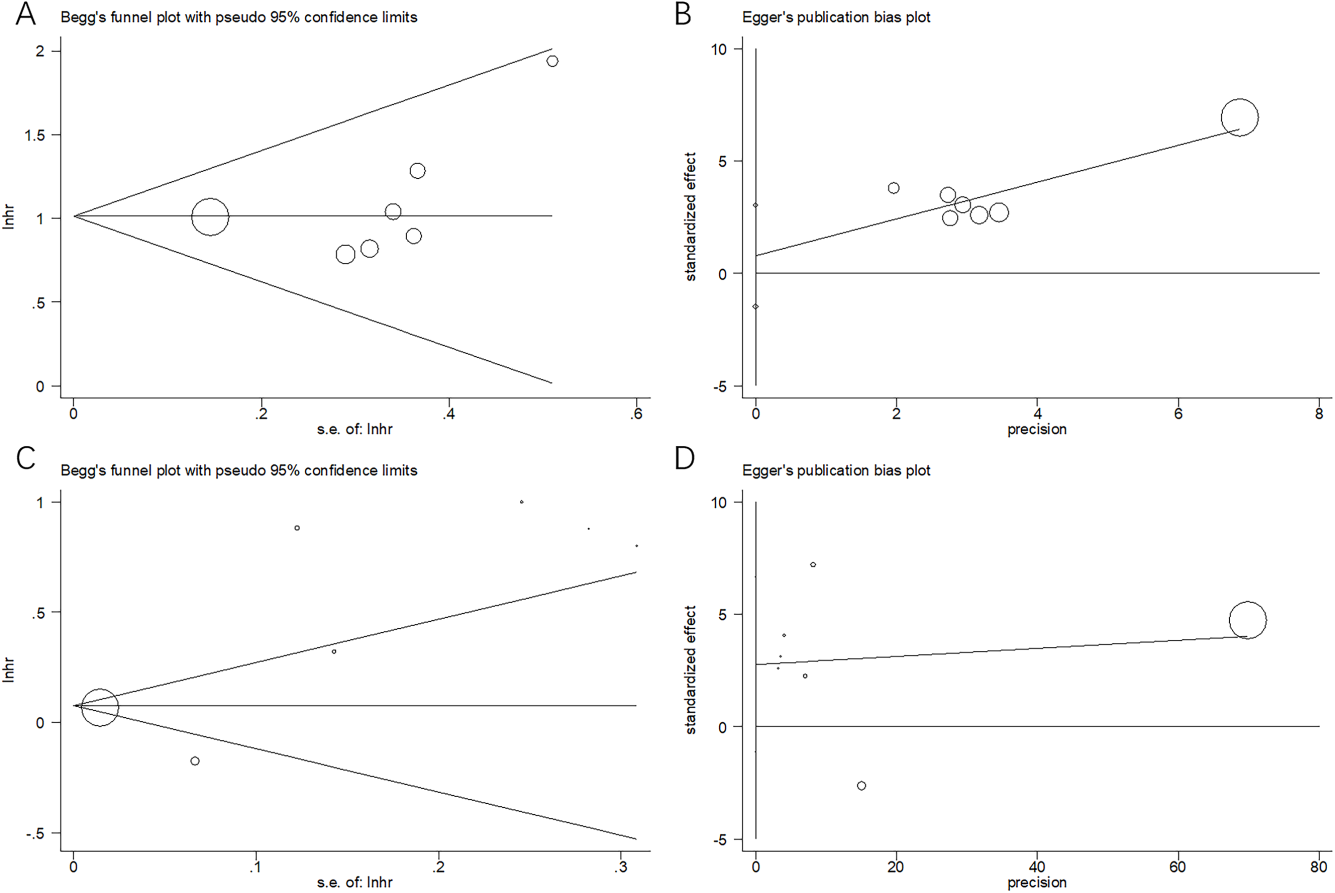
Assessment of publication bias: **(A)** Begg’s test for OS, *p* = 0.072; **(B)** Egger’s test for OS, *p* = 0.415; **(C)** Begg’s test for PFS, *p* = 0.548; and **(D)** Egger’s test for PFS, *p* = 0.126.

Begg’s and Egger’s tests were used to assess publication bias. Forest plots were symmetrical, revealing no apparent publication bias for OS (*p* = 0.072 and 0.415 for Begg’s and Egger’s tests, respectively) or PFS (*p* = 0.548 and 0.126 for Begg’s and Egger’s tests, respectively) in this meta-analysis (**Figure 5**).

## Discussion

FAR has been widely studied for its prognostic value in gynecological cancers, although previous findings have been inconsistent. This meta-analysis incorporated data from 10 studies involving 1,902 patients (17–26) to clarify the prognostic significance of FAR in gynecological cancers. Our results revealed a significant correlation between elevated FAR and poorer OS and PFS. Moreover, the prognostic value of FAR remained consistent across various subgroup analyses. Sensitivity and publication bias analyses supported the credibility of these findings. Overall, elevated FAR appears to be a strong predictor of both short- and long-term survival outcomes in gynecological cancers. To our knowledge, this meta-analysis is the first to comprehensively evaluate the prognostic value of FAR in this context.

FAR is calculated based on the levels of fibrinogen and albumin. An elevated FAR may result from increased fibrinogen levels, decreased albumin levels, or both. Although the precise mechanisms underlying FAR’s prognostic value in gynecological cancers remain to be fully elucidated, several explanations have been proposed. First, studies have shown that fibrinogen levels can rise under various pathophysiological conditions, including tumors, surgeries, infections, inflammations, or trauma (29). Fibrinogen is primarily produced by the liver and, in some cases, by malignant tumor cells. It is released into the bloodstream, with this release being amplified by systemic inflammatory responses (30). Moreover, fibrinogen promotes the production of inflammatory cytokines and is regarded as a reliable indicator of systemic inflammation (31). It also contributes to tumor cell coagulation, enhancing cancer cell survival and adhesion, thereby facilitating metastasis (32). Second, serum albumin is widely considered a marker of immune and nutritional status, with low albumin levels linked to poorer postoperative outcomes and cachexia in patients with cancer (33). Albumin, synthesized by liver parenchyma cells, serves as an indicator of liver reserve function and is essential for antioxidant defense, endothelial stability, and immune regulation (34). Serum albumin levels are positively correlated to body mass index and nutritional status, independent of systemic inflammation and liver function (35). Low albumin levels are linked to an increased risk of malnutrition, reduced OS, and elevated inflammatory markers such as interleukin-6 and C-reactive protein (36). Therefore, FAR represents a rational prognostic marker based on the biological roles of fibrinogen and albumin.

Notably, significant heterogeneity was observed in the PFS analysis (**Table 3**). This heterogeneity may be attributed to several factors. First, the results of the included studies on PFS were inconsistent. For instance, the study by Onoprienko (24) reported results that contradicted those of other studies (17–23, 25, 26) (**Figure 3**), which may be a major source of the heterogeneity. Second, despite this variability, sensitivity analysis and publication bias tests confirmed the reliability of the overall PFS results (**Figures 4**, **5**).

The cutoff values reported in the included studies ranged from 0.097 to 0.13, with a median of 0.11. Therefore, we applied 0.11 as the cutoff value in the subgroup analyses for OS and PFS. The results indicated that FAR ≥ 0.11 consistently demonstrated significant prognostic value for both outcomes (**Tables 2**, **3**). Based on these findings, we recommend 0.11 as the standard cutoff value for FAR in future studies on gynecological cancers.

Notably, literature assessment and decision-making in systematic reviews are crucial processes (37). Typically, three reviewers are involved in such studies, with decisions made by majority vote. In the present meta-analysis, two independent investigators were involved in data extraction. Two investigators (Y.C. and J.Z.) independently collected data from eligible articles following the PRISMA guidelines, and any disagreements were resolved through discussion until a consensus was reached. Although the PRISMA guidelines were strictly followed, the involvement of only two reviewers is considered a limitation of this study.

Recently, many studies reported that FAR can serve as a prognostic indicator for various cancers based on meta-analyses (38–40). In a meta-analysis by Zhang et al. involving 5,088 cases, a high FAR was significantly associated with poor OS and worse disease-free survival (DFS) in malignant tumors (38). Similarly, a meta-analysis by Wang et al. indicated that elevated FAR was linked to poor OS and DFS in breast cancer and was closely related to multiple indicators of tumor progression (39). Li et al. conducted a meta-analysis of 19 studies and concluded that a higher FAR was markedly associated with worse survival outcomes in patients with cancer (40). The findings of our study are consistent with these results, further supporting the prognostic significance of FAR in various cancer types.

This study has several limitations that should be acknowledged. First, many of the included studies were conducted in China, which may limit the generalizability of our findings to Chinese patients with gynecological cancers. Second, all eligible studies were retrospective in design, and the possibility of selection bias cannot be excluded. Third, the threshold for FAR varied across studies, leading to potential inconsistency in prognostic evaluation. Therefore, large-scale, multicenter prospective clinical studies are needed to validate these findings.

## Conclusion

This meta-analysis demonstrates that elevated FAR is significantly associated with poorer OS and inferior in patients with gynecological cancers. FAR may serve as a promising prognostic biomarker in clinical settings.

## Data Availability

The original contributions presented in the study are included in the article/**Supplementary Material**. Further inquiries can be directed to the corresponding author.
