# Supplementary material for: Prognostic role of fibrinogen-to-albumin ratio in patients with gynecological cancers: a meta-analysis
Source: Front Oncol. 2025 Jul 3;15:1580940. doi: 10.3389/fonc.2025.1580940 (PMC12267011; doi:10.3389/fonc.2025.1580940)
Supplement: Supplementary file 1 [file DataSheet1.docx]

**Supplemental file 1. The detailed search strategies for each database.**

**1. Search strategies for PubMed:**

Search: (albumin-to-fibrinogen or albumin/fibrinogen or fibrinogen-to-albumin or fibrinogen/albumin) and (gynecological cancer OR cervical cancer OR ovarian cancer OR endometrial cancer OR gynecological carcinoma OR cervical carcinoma OR ovarian carcinoma OR endometrial carcinoma OR gynecological neoplasm or vulvar cancer or vaginal cancer) Sort by: Most Recent

("albumin-to-fibrinogen"[All Fields] OR "albumin fibrinogen"[All Fields] OR "fibrinogen-to-albumin"[All Fields] OR "fibrinogen albumin"[All Fields]) AND ((("gynaecologic"[All Fields] OR "gynecologic"[All Fields] OR "gynecologically"[All Fields] OR "gynecology"[MeSH Terms] OR "gynecology"[All Fields] OR "gynaecological"[All Fields] OR "gynecological"[All Fields]) AND ("cancer s"[All Fields] OR "cancerated"[All Fields] OR "canceration"[All Fields] OR "cancerization"[All Fields] OR "cancerized"[All Fields] OR "cancerous"[All Fields] OR "neoplasms"[MeSH Terms] OR "neoplasms"[All Fields] OR "cancer"[All Fields] OR "cancers"[All Fields])) OR ("uterine cervical neoplasms"[MeSH Terms] OR ("uterine"[All Fields] AND "cervical"[All Fields] AND "neoplasms"[All Fields]) OR "uterine cervical neoplasms"[All Fields] OR ("cervical"[All Fields] AND "cancer"[All Fields]) OR "cervical cancer"[All Fields]) OR ("ovarian neoplasms"[MeSH Terms] OR ("ovarian"[All Fields] AND "neoplasms"[All Fields]) OR "ovarian neoplasms"[All Fields] OR ("ovarian"[All Fields] AND "cancer"[All Fields]) OR "ovarian cancer"[All Fields]) OR ("endometrial neoplasms"[MeSH Terms] OR ("endometrial"[All Fields] AND "neoplasms"[All Fields]) OR "endometrial neoplasms"[All Fields] OR ("endometrial"[All Fields] AND "cancer"[All Fields]) OR "endometrial cancer"[All Fields]) OR (("gynaecologic"[All Fields] OR "gynecologic"[All Fields] OR "gynecologically"[All Fields] OR "gynecology"[MeSH Terms] OR "gynecology"[All Fields] OR "gynaecological"[All Fields] OR "gynecological"[All Fields]) AND ("carcinoma"[MeSH Terms] OR "carcinoma"[All Fields] OR "carcinomas"[All Fields] OR "carcinoma s"[All Fields])) OR (("cervic"[All Fields] OR "cervicals"[All Fields] OR "cervices"[All Fields] OR "neck"[MeSH Terms] OR "neck"[All Fields] OR "cervical"[All Fields] OR "uterine cervicitis"[MeSH Terms] OR ("uterine"[All Fields] AND "cervicitis"[All Fields]) OR "uterine cervicitis"[All Fields] OR "cervicitis"[All Fields]) AND ("carcinoma"[MeSH Terms] OR "carcinoma"[All Fields] OR "carcinomas"[All Fields] OR "carcinoma s"[All Fields])) OR ("ovarian neoplasms"[MeSH Terms] OR ("ovarian"[All Fields] AND "neoplasms"[All Fields]) OR "ovarian neoplasms"[All Fields] OR ("ovarian"[All Fields] AND "carcinoma"[All Fields]) OR "ovarian carcinoma"[All Fields]) OR ("endometrial neoplasms"[MeSH Terms] OR ("endometrial"[All Fields] AND "neoplasms"[All Fields]) OR "endometrial neoplasms"[All Fields] OR ("endometrial"[All Fields] AND "carcinoma"[All Fields]) OR "endometrial carcinoma"[All Fields]) OR (("gynaecologic"[All Fields] OR "gynecologic"[All Fields] OR "gynecologically"[All Fields] OR "gynecology"[MeSH Terms] OR "gynecology"[All Fields] OR "gynaecological"[All Fields] OR "gynecological"[All Fields]) AND ("neoplasm s"[All Fields] OR "neoplasms"[MeSH Terms] OR "neoplasms"[All Fields] OR "neoplasm"[All Fields])) OR ("vulvar neoplasms"[MeSH Terms] OR ("vulvar"[All Fields] AND "neoplasms"[All Fields]) OR "vulvar neoplasms"[All Fields] OR ("vulvar"[All Fields] AND "cancer"[All Fields]) OR "vulvar cancer"[All Fields]) OR ("vaginal neoplasms"[MeSH Terms] OR ("vaginal"[All Fields] AND "neoplasms"[All Fields]) OR "vaginal neoplasms"[All Fields] OR ("vaginal"[All Fields] AND "cancer"[All Fields]) OR "vaginal cancer"[All Fields]))

Translations

gynecological: "gynaecologic"[All Fields] OR "gynecologic"[All Fields] OR "gynecologically"[All Fields] OR "gynecology"[MeSH Terms] OR "gynecology"[All Fields] OR "gynaecological"[All Fields] OR "gynecological"[All Fields]

cancer: "cancer's"[All Fields] OR "cancerated"[All Fields] OR "canceration"[All Fields] OR "cancerization"[All Fields] OR "cancerized"[All Fields] OR "cancerous"[All Fields] OR "neoplasms"[MeSH Terms] OR "neoplasms"[All Fields] OR "cancer"[All Fields] OR "cancers"[All Fields]

cervical cancer: "uterine cervical neoplasms"[MeSH Terms] OR ("uterine"[All Fields] AND "cervical"[All Fields] AND "neoplasms"[All Fields]) OR "uterine cervical neoplasms"[All Fields] OR ("cervical"[All Fields] AND "cancer"[All Fields]) OR "cervical cancer"[All Fields]

ovarian cancer: "ovarian neoplasms"[MeSH Terms] OR ("ovarian"[All Fields] AND "neoplasms"[All Fields]) OR "ovarian neoplasms"[All Fields] OR ("ovarian"[All Fields] AND "cancer"[All Fields]) OR "ovarian cancer"[All Fields]

endometrial cancer: "endometrial neoplasms"[MeSH Terms] OR ("endometrial"[All Fields] AND "neoplasms"[All Fields]) OR "endometrial neoplasms"[All Fields] OR ("endometrial"[All Fields] AND "cancer"[All Fields]) OR "endometrial cancer"[All Fields]

gynecological: "gynaecologic"[All Fields] OR "gynecologic"[All Fields] OR "gynecologically"[All Fields] OR "gynecology"[MeSH Terms] OR "gynecology"[All Fields] OR "gynaecological"[All Fields] OR "gynecological"[All Fields]

carcinoma: "carcinoma"[MeSH Terms] OR "carcinoma"[All Fields] OR "carcinomas"[All Fields] OR "carcinoma's"[All Fields]

cervical: "cervic"[All Fields] OR "cervicals"[All Fields] OR "cervices"[All Fields] OR "neck"[MeSH Terms] OR "neck"[All Fields] OR "cervical"[All Fields] OR "uterine cervicitis"[MeSH Terms] OR ("uterine"[All Fields] AND "cervicitis"[All Fields]) OR "uterine cervicitis"[All Fields] OR "cervicitis"[All Fields]

carcinoma: "carcinoma"[MeSH Terms] OR "carcinoma"[All Fields] OR "carcinomas"[All Fields] OR "carcinoma's"[All Fields]

ovarian carcinoma: "ovarian neoplasms"[MeSH Terms] OR ("ovarian"[All Fields] AND "neoplasms"[All Fields]) OR "ovarian neoplasms"[All Fields] OR ("ovarian"[All Fields] AND "carcinoma"[All Fields]) OR "ovarian carcinoma"[All Fields]

endometrial carcinoma: "endometrial neoplasms"[MeSH Terms] OR ("endometrial"[All Fields] AND "neoplasms"[All Fields]) OR "endometrial neoplasms"[All Fields] OR ("endometrial"[All Fields] AND "carcinoma"[All Fields]) OR "endometrial carcinoma"[All Fields]

gynecological: "gynaecologic"[All Fields] OR "gynecologic"[All Fields] OR "gynecologically"[All Fields] OR "gynecology"[MeSH Terms] OR "gynecology"[All Fields] OR "gynaecological"[All Fields] OR "gynecological"[All Fields]

neoplasm: "neoplasm's"[All Fields] OR "neoplasms"[MeSH Terms] OR "neoplasms"[All Fields] OR "neoplasm"[All Fields]

vulvar cancer: "vulvar neoplasms"[MeSH Terms] OR ("vulvar"[All Fields] AND "neoplasms"[All Fields]) OR "vulvar neoplasms"[All Fields] OR ("vulvar"[All Fields] AND "cancer"[All Fields]) OR "vulvar cancer"[All Fields]

vaginal cancer: "vaginal neoplasms"[MeSH Terms] OR ("vaginal"[All Fields] AND "neoplasms"[All Fields]) OR "vaginal neoplasms"[All Fields] OR ("vaginal"[All Fields] AND "cancer"[All Fields]) OR "vaginal cancer"[All Fields]

**2. Search strategies for Web of Science:**

(albumin-to-fibrinogen or albumin/fibrinogen or fibrinogen-to-albumin or fibrinogen/albumin) and (gynecological cancer OR cervical cancer OR ovarian cancer OR endometrial cancer OR gynecological carcinoma OR cervical carcinoma OR ovarian carcinoma OR endometrial carcinoma OR gynecological neoplasm or vulvar cancer or vaginal cancer) (title/abstract)

**3. Search strategies for Embase:**

(albumin-to-fibrinogen or albumin/fibrinogen or fibrinogen-to-albumin or fibrinogen/albumin) and (gynecological cancer OR cervical cancer OR ovarian cancer OR endometrial cancer OR gynecological carcinoma OR cervical carcinoma OR ovarian carcinoma OR endometrial carcinoma OR gynecological neoplasm or vulvar cancer or vaginal cancer)

**4.** **Search strategies for Cochrane Library:**

(albumin-to-fibrinogen or albumin/fibrinogen or fibrinogen-to-albumin or fibrinogen/albumin) and (gynecological cancer OR cervical cancer OR ovarian cancer OR endometrial cancer OR gynecological carcinoma OR cervical carcinoma OR ovarian carcinoma OR endometrial carcinoma OR gynecological neoplasm or vulvar cancer or vaginal cancer) in Title Abstract Keyword

**5. Search strategies for China National Knowledge Infrastructure (CNKI):**

FAR and 宫颈

FAR and 子宫

FAR and 卵巢

FAR and 外阴

FAR and 阴道
